# Introduction of a Comprehensive Diagnostic and Interdisciplinary Management Approach in Haematological Patients with Mucormycosis: A Pre and Post-Intervention Analysis

**DOI:** 10.3390/jof6040268

**Published:** 2020-11-08

**Authors:** Malene Risum, Jannik Helweg-Larsen, Søren Lykke Petersen, Peter Kampmann, Ulrik Malthe Overgaard, Daniel El Fassi, Ove Juul Nielsen, Mette Brabrand, Niclas Rubek, Lars Munksgaard, Marianne Tang Severinsen, Bendt Nielsen, Jan Berg Gertsen, Åsa Gylfe, Ulla Hjort, Angeliki Vourtsi, Rasmus Krøger Hare, Maiken Cavling Arendrup

**Affiliations:** 1 Unit of Mycology, Statens Serum Institut, 2300 Copenhagen, Denmark; mlri@ssi.dk (M.R.); rmj@ssi.dk (R.K.H.); 2 Department of Infectious Diseases, Copenhagen University Hospital, Rigshospitalet, 2100 Copenhagen, Denmark; Jannik.Helweg-Larsen@regionh.dk; 3 Department of Haematology, Copenhagen University Hospital, Rigshospitalet, 2100 Copenhagen, Denmark; Soeren.Lykke.Petersen@regionh.dk (S.L.P.); Peter.Kampmann@regionh.dk (P.K.); ulrik.malthe.overgaard@regionh.dk (U.M.O.); Ove.Juul.Nielsen@regionh.dk (O.J.N.); 4 Department of Haematology, Herlev and Gentofte Hospital, 2730 Herlev, Denmark; daniel.el.fassi.01@regionh.dk; 5 Department of Clinical Medicine, University of Copenhagen, 2200 Copenhagen, Denmark; 6 Department of Haematology, Odense University Hospital, 5000 Odense, Denmark; Mette.Brabrand@rsyd.dk; 7 Department of Otorhinolaryngology, Rigshospitalet, 2100 Copenhagen, Denmark; Niclas.Rubek@regionh.dk; 8 Department of Haematology, Zealand University Hospital, 4000 Roskilde, Denmark; larmu@regionsjaelland.dk; 9 Department of Haematology, Aalborg University Hospital, 9100 Aalborg, Denmark; m.severinsen@rn.dk; 10 Department of Clinical Medicine, Aalborg University Hospital, 9100 Aalborg, Denmark; 11 Department of Haematology, Aarhus University Hospital, 8200 Aarhus, Denmark; bendt.nielsen@aarhus.rm.dk; 12 Department of Clinical Microbiology, Aarhus University Hospital, 8200 Aarhus, Denmark; Jan.Berg.Gertsen@rm.dk; 13 Department of Clinical Microbiology, Umeå University, 901 85 Umeå, Sweden; asa.gylfe@umu.se; 14 Department of Infectious Diseases, Aalborg University Hospital, 9100 Aalborg, Denmark; ulh@rn.dk; 15 Department of Haematology, Norrlands Universitetssjukhus, 907 37 Umeå, Sweden; Angeliki.Vourtsi@regionvasterbotten.se; 16 Department of Clinical Microbiology, Rigshospitalet, 2100 Copenhagen, Denmark

**Keywords:** Mucorales, mucormycosis, haematology, neutropenia and mortality

## Abstract

Mucormycosis is a life threatening infection in patients with haematological disease. We introduced a Mucorales-PCR and an aggressive, multidisciplinary management approach for mucormycosis during 2016–2017 and evaluated patient outcomes in 13 patients diagnosed and treated in 2012–2019. Management principle: repeated surgical debridement until biopsies from the resection margins were clean as defined by negative Blankophor microscopy, Mucorales-PCR (both reported within 24 h), and cultures. Cultured isolates underwent EUCAST E.Def 9.3.1 susceptibility testing. Antifungal therapy (AFT) (mono/combination) combined with topical AFT (when possible) was given according to the minimal inhibitory concentration (MIC), severity of the infection, and for azoles, specifically, it was guided by therapeutic drug monitoring. The outcome was evaluated by case record review. All patients underwent surgery guided by diagnostic biopsies from tissue and resection margins (195 samples in total). Comparing 2012–2015 and 2016–2019, the median number of patients of surgical debridements was 3 and 2.5 and of diagnostic samples: microscopy/culture/PCR was 3/3/6 and 10.5/10/10.5, respectively. The sensitivity of microscopy (76%) and Mucorales-PCR (70%) were similar and microscopy was superior to that of culture (53%; *p* = 0.039). Initial systemic AFT was liposomal amphotericin B (*n* = 12) or posaconazole (*n* = 1) given as monotherapy (*n* = 4) or in combination with isavuconazole/posaconazole (*n* = 3/6) and terbinafine (*n* = 3). Nine patients received topical amphotericin B. All received isavuconazole or posaconazole consolidation therapy (*n* = 13). Mucormycosis related six month mortality was 3/5 in 2012–2015 and 0/7 patients in 2016–2019 (one patient was lost for follow-up). Implementation of combination therapy (systemic+topical AFT/combination systemic AFT) and aggressive surgical debridement guided by optimised diagnostic tests may improve the outcome of mucormycosis in haematologic patients.

## 1. Introduction

Mucormycosis is a rare, angio-invasive fungal infection [1]. The incidence is difficult to estimate since systematic surveillance is not performed and cases may be classified as an unspecified invasive fungal infection in the absence of definitive microbiological identification. The overall mortality is almost 100% if untreated [2]. The most common genera involved are *Rhizopus*, *Mucor, Rhizomucor*, and *Lichtheimia* [3].

In industrial countries, the major predisposing factor for mucormycosis is haematological malignancy [1,4,5,6]. The most common manifestations are rhino-orbital-cerebral-mucormycosis (ROCM) and pulmonary mucormycosis [1,5,7]. Pulmonary mucormycosis is the most frequent manifestation in haematological patients [7,8,9,10,11]. Predisposing risk factors are prolonged and severe neutropenia, high-dose corticosteroids, and iron overload (IOL) [12].

Factors that impact survival are the underlying condition, surgical and antifungal treatment, and site of infection [1,2,7,10]. Two comprehensive reviews on mucormycosis reported an overall mortality of 54% and 46%, respectively [2,13]. However, only 15.8% and 32% of the included patients had haematological disease [2,13]. Disseminated infection is associated with haematological disease [10,13] and carries the highest mortality (55–80%) [2]. Mortality is particularly high in relation to allogeneic haematopoietic stem cell transplantation (allo-HSCT), in which 100 days mortality is 71% [14]. Early surgery and targeted antifungal treatment is crucial. A delay of six days or more to the initiation of therapy has been shown to increase mortality in haematological patients receiving amphotericin B [15].

The recent global guideline recommends CT scanning, microscopy with fluorescent brightener, and culture for the diagnosis. Although typical microscopic findings are irregular broad pauci-septate 90° branching hyphae, artefacts may confuse the interpretations, particularly for technicians without specific mycology expertise. Therefore, confirmation of microscopy is advised through in situ immunohistochemistry or PCR, where these tests are available and with the caveat that no commercial standardised PCR assays are available and the diagnostic performance of in house tests may vary [16]. Of note, no comments are offered regarding the use of these tests for guiding the need for repeated surgery and fluorescent brightener is not universally implemented in clinical microbiology laboratories.

There are no validated antifungal breakpoints for Mucorales [3,17]. Amphotericin B is in vitro active against most Mucorales species, but with high MICs against *Cunninghamella* species [3]. Isavuconazole and posaconazole have also shown in vivo and in vitro activity with MICs similar to those found for *Aspergillus*, except against *Mucor circinelloides* [18]. Itraconazole has some activity, but is not considered a first line therapy [19]. In contrast, fluconazole and voriconazole have no clinical activity against Mucorales [3].

Current guidelines from ECIL-6, ESCMID, and ECMM (European Confederation on Infection in Leukaemia, European Society of Clinical Microbiology and Infectious Disease, and European Confederation of Medical Mycology) recommend surgical debridement and high dose liposomal amphotericin B (5 mg/kg) together with control of the underlying predisposing condition [16,17,20]. If the CNS is involved, a dosage of liposomal amphotericin B of 10 mg/kg initially is recommended [16,20]. First line treatment with high dose liposomal amphotericin B is strongly recommended, while isavuconazole and posaconazole are recommended with moderate strength. Both triazoles are strongly recommended as salvage treatments [16] and isavuconazole has been approved in the USA as an alternative to liposomal amphotericin B as a first line treatment in mucormycosis. Its licence in Europe is reserved for mucormycosis in patients for whom amphotericin B is inappropriate. Hyperbaric oxygen can be considered as a supportive treatment, whereas deferasirox use in combination with amphotericin B has been associated with increased mortality and should be avoided [16,17,20,21].

The outcome of mucormycosis remains poor despite advances in the antifungal armamentarium. We sought to improve the outcome through an intensified multidisciplinary management approach gradually introduced during 2016–2017 and consisting of surgical revision guided by rapid diagnostics and aggressive combination antifungal therapy. We investigated the survival rate before and after the intervention in a series of 13 patients with proven mucormycosis in 2012 to June 2019 and found no mucormycosis related deaths since 2015.

## 2. Materials and Methods

This is a case-series based review including all patients with proven mucormycoses from January 2012 to June 2019 for whom mycology advice or diagnostic investigations were requested at the national reference centre for mycology at Statens Serum Institut (SSI). The reference laboratory serves the entire country with diagnostic tests and therapeutic advice as well as species identification and reference susceptibility testing of referred isolates. The current study therefore probably includes the vast majority of proven mucormycosis cases during the study period in Denmark, although we cannot exclude that undiagnosed cases or non-proven cases in patients beyond therapeutic reach and for whom diagnostics or consultation were deemed unnecessary were missed in the current period. One case involved a patient from Sweden (#12) for whom the SSI was consulted concerning diagnostics and management. One patient’s case has been published previously [22].

### 2.1. Laboratory Investigations

Biopsies were examined by Blankophor microscopy (SSI Diagnostica, Hillerød, Denmark), cultured at YGC medium (Oxoid A/S Thermo Scientific, Roskilde, Denmark) and since 2017, investigated by an in-house Mucorales specific PCR (see below). For one laboratory in Sweden, Sabouraud Dextrose Agar (LabM, Lancashire, UK) supplemented with yeast extract 2.5 g/L (Oxoid, Roskilde, Denmark) and chloramphenicol 50 µg/L was used. Species identification of cultured isolates was performed by micro- and macro-morphology, supplemented by carbon assimilation using ATB ID32C (bioMérieux, Marcy l’Etoile, France) [23], sequencing of the internal transcribed spacer (ITS) [24], and/or 18 S analysis as necessary [25]. The EUCAST E.Def 9.3.1 reference method was used for susceptibility testing [26]. For culture negative samples, the species identification was obtained by direct ITS PCR and sequencing [24], 18 S analysis [25], and/or Mucorales specific PCR (described below). Thus, in all cases, the involved species identification was molecularly confirmed.

### 2.2. Mucorales Diagnostic PCRs

(TaqMan) were introduced in 2017 and adopted with slight modifications from those described by Salehi et al. [27]. One test detects *Rhizopus microsporus, Rhizopus arrhizus*, and *Mucor* spp. A second test detects *Lichtheimia* spp. and *Rhizomucor* spp. DNA extraction Qiacube (Qiagen, Copenhagen, Denmark) standard protocol is for blood and tissue samples, with up to 200 μL input volume and elution in 100 μL. Formalin fixed paraffin-embedded tissue samples were processed using Neo-clear (Sigma- Aldrich, Søborg, Denmark) followed by the protocol as described above. BALs were bead beaten and DNA extracted on the NucliSENS easyMAG (Biomérieux, Ballerup, Denmark) platform with up to 1 mL input volume and elution in 60 μL. PCR analyses were performed on the Applied Biosystems 7500 and since mid-2018, the Quantstudio 5 thermocyclers (Roskilde, Thermo Fisher Scientific, Denmark). Positive and negative template controls were applied, but not PCR inhibition or DNA extraction controls. TaqMan Multiplex Master Mix (polymerase, buffer and dNTPs) as well as primers and probes were acquired from Thermo Fisher Scientific (Glasgow, UK) except for a one locked nucleic acid probe (*R. microsporus*) from IDT (Integrated DNA Technologies, München-Flughafen, Germany).

### 2.3. Optimised Clinical Management Principles Were As Follows

Aggressive primary surgery with complete resection of tissue and bone necrosis if possible, guided by microscopy. Supporting imaging was performed in relevant cases. Consecutive debridements were repeated often several times a week until the resection margins were clean. This was defined by negative Blankophor microscopy, Mucorales specific PCR, and cultures of biopsies. Early diagnosis was possible by means of same day Blankophor microscopy and same/next day Mucorales-PCR results. Targeted antifungal therapy combined with topical therapy (when possible) was given according to the MIC (mg/L) and severity of the infection. High trough azole levels above MIC were ensured by target drug monitoring (TDM) [28]. Reconstruction with free flaps should be considered early in cases of exposed bone to ensure optimal effect of the systemic antifungal treatment.

Fungal free survival and further clinical data were evaluated by case record review and history provided by the treating physicians at the time of the ongoing fungal infection. Permission for data review was given according to Danish quality-based research regulations.

### 2.4. Data Management

Positive rates for the diagnostic tests performed within the first 7 days were compared pairwise using Fisher’s exact test (two-sided) with *p* value < 0.05 regarded significant using GraphPad Prism 8.3.0 (Prism – GraphPad, San Diego, CA, USA).

Overall, three, six, and 12 month survival rates were determined and three and six month mucormycosis related deaths specifically were determined in the two periods 2012–2015 and 2016–2018.

## 3. Results

Thirteen cases of proven mucormycosis in patients with haematological disease (January 2012 to June 2019) were identified and reviewed (Table 1). The median age was 36 years (range: 3–70 years). The majority were males (8/13). Underlying diseases were aplastic anaemia (*n* = 4), acute myeloid leukaemia (AML) (*n* = 3), acute lymphoblastic leukaemia (ALL) (*n* = 2), acute lymphoblastic T-cell lymphoma (*n* = 1), Burkitt lymphoma (*n* = 1), diffuse large B-cell lymphoma (*n* = 1), and myelodysplastic syndrome (MDS) (*n* = 1). A detailed case review is available in the Supplementary Materials. The involved species were *R. microsporus* (*n* = 6), *R. arrhizus* (*n* = 2), *Rhizomucor pusillus* (*n* = 2), *Lichtheimia corymbifera* (*n* = 1), *Lichtheimia ramosa* (*n* = 1), and *Circinella muscae* (*n* = 1). The localizations of the infection were the sinuses (*n* = 7), brain (*n* = 3), lung (*n* = 3), liver (*n* = 1), skin (*n* = 1), vertebrae (*n* = 1), and a sub-diaphragmatic abscess (*n* = 1). Three patients had infections involving neighbour or non-contiguous sites of infection, including one patient with infection involving three separate sites. Two patients with sinus involvement had CNS involvement. Three of the patients with sinus involvement reported marijuana smoking. Five patients had received posaconazole as mould prophylaxis for more than one week prior to the infection (excluding one patient with only three days of posaconazole before the symptoms debut).

The results of diagnostic tests, species identification, and susceptibility data are summarised in Table 2 and Table 3. Overall, the fungal infections were confirmed by a positive microscopy in 12/13 patients, by culture in 9/13 patients, and by molecular diagnostic tests in 8/10 patients. The median total number of samples per patient sent for culture was 3 (range 1–18) in 2012–2015 and 10.5 (range 1–55) in 2016–2019. Similarly, the figures for microscopy were 3 (range 0–15) and 10.5 (range 2–60) in 2012–2015 and 2016–2019, respectively. Finally, the number of molecular tests performed was 6 (range 0–24) and 10 (range 0–67) per patient in 2012–2015 and 2016–2019, respectively (Table 2). Assuming that samples taken early are more representative for the diagnostic performance than later during antifungal therapy, which included control samples taken from presumed healthy tissue margins, we compared the positive rates including only samples taken within the first 7 days (Table 3). The positive rate was similar for microscopy 76% (29/38 samples) (95% CI: 61–87%), 18 S PCR 77% (10/13 samples) (95% CI: 50–92%), and Mucorales-PCR 70% (14/20) (95% CI: 48–86%) and higher than that for culture 53% (23/43) (95% CI: 39–67%) for culture and ITS PCR 53% (10/19) (95% CI: 32–73%). The difference was statistically significant for microscopy compared to culture (*p* = 0.039). When comparing samples taken from the same date, which underwent microscopy and culture simultaneously at both the local and reference laboratories, none were positive (0/37) at direct microscopy at the local laboratory (not using Blankophor staining), while 54% (21/39) were positive with pauci-septate hyphae at the reference laboratory using Blankophor staining. The culture positive rate was similar to 10% (5/49) at the local laboratory and 12% (5/42) at the reference laboratory.

All patients except one since 2016 underwent multiple surgical interventions up to 47 interventions (extending from inspection to larger resections in one patient). After the introduction of Mucorales-PCR, four out of five patients had species identification confirmed by Mucorales-PCR and three patients had continuously Mucorales-PCR performed together with microscopy in relation to the surgical resections. Over the years, patients have been diagnosed and treated in an increasingly closer collaboration between treating physicians and the mycology unit, resulting in an increasing number of diagnostic tests and an earlier diagnosis with species identification possible within 24 h due to the Mucorales-PCR (Table 2).

Initial systemic targeted treatment was liposomal amphotericin B in 12 out of 13 patients of whom nine received this in combination with either posaconazole or isavuconazole (Table 4). Three patients received dosages of >5 mg/kg liposomal amphotericin B initially due to suspicion of CNS involvement. Three patients also received terbinafine and all received an azole as consolidation therapy. Nine out of 13 patients received topical amphotericin B, of whom two patients received amphotericin B intrathecally. The number of patients receiving topical antifungal treatment was 3/5 in 2012–2015 and 6/8 in 2016–2019. For intrathecal use, one patient was treated with amphotericin B deoxycholate 0.5 mg/dose daily or three times a week intrathecally (#2). A second patient initially received liposomal amphotericin B 10 mg/dose, which was complicated by severe painful arachnoiditis (#13) and thereafter, amphotericin B deoxycholate 0.1–0.2 mg/dose administrated during 20 min every other day and postoperatively, amphotericin B 2.5 mg twice intrathecally [29].

Choice and dose of systemic antifungal treatment was adjusted according to species, susceptibility pattern, anatomic localisation (local disease vs. disseminated and CNS involvement), and the TDM result when measured. TDM was performed in 10 patients of whom seven received posaconazole and four isavuconazole (patient #8 received posaconazole and isavuconazole sequentially). TDM was individualized according to the fungal MICs, keeping a level clearly above the MIC, and according to the severity and clinical course.

The overall three, six, and 12 month survival rates were 83% (10/12), 67% (8/12), and 58% (7/12), respectively (Table 5), excluding one patient who was lost for follow-up one week after diagnosis. The three and six months’ mucormycosis related mortality rates were 17% (2/12) and 25% (3/12), respectively. The overall six month survival rates were 1/3 and 7/9 among patients with and without CNS involvement. The six month survival rate was 6/9 for patients receiving combined systemic antifungal treatment and 2/3 for those who received monotherapy. For patients receiving topical antifungal treatment, 7/8 were alive after six months, as opposed to 1/4 for those who did not receive topical antifungal treatment. Finally, six month mucormycosis related deaths occurred in 3/5 and 0/7 patients in 2012–2015 and 2016-June 2019, respectively. In seven out of 13 patients, the haematological treatment was suspended during mucormycosis treatment, which affected the process of treatment of underlying disease in 6 out of 13 patients. This led to haematological relapse in two further patients after three and six months who were cured for mucormycosis (Table 5).

## 4. Discussion

We report 13 cases with proven Mucorales infection diagnosed during 2012–18 and with an overall three, six, and 12 month survival rate of 83%, 67%, and 58%, respectively, with no mucormycosis related deaths after 2015. A multidisciplinary aggressive management approach including a new in house Mucorales-PCR test was introduced during 2016 and 2017, leading to a three times doubling of diagnostic tests per patient for diagnosis and guidance of surgery. All patients diagnosed during 2016–2018 survived the infection and none of the patients with sinus mucormycosis progressed to orbital or CNS involvement. All but one of the patients who received topical antifungal treatment survived the infection, which suggests a potential survival benefit. Although numbers are low, we speculate that the apparently improved outcome compared to patients diagnosed earlier may be a result of a closer collaboration between specialties, leading to early diagnosis and diagnostics driven surgical treatment. Microscopy and Mucorales-PCR enable a more precise guidance of surgery, leading to a more favourable mucormycosis outcome in close collaboration between the haematologist, infectious disease physician, and surgeon, with focus on topical treatment in relation to surgery.

Combined surgery and antifungal treatment have previously been associated with a better outcome of mucormycosis in several studies [2,19,30]. However, surgical debridement for mucormycosis is often mutilating, especially in haematological patients with profound thrombocytopenia. These factors necessitate a careful risk assessment, balancing the risks and cosmetic consequences associated with the surgery with the certainty of the diagnosis, amount of affected tissue, and likely outcome. Manesh et al. found a higher mortality in haematological patients and ascribed it to lower rates of surgical debridements [31]. Vironneau et al. found that patients with ROCM not eligible for surgery were more likely to die from their mucormycosis and that local control with repeated and wide surgery had an impact on survival [32]. Rapid diagnostics allow early antifungal therapy and surgery and consequently may limit the extent and number of subsequent surgical revisions, caused by the aggressive angio invasive growth of Mucorales. Blankophor microscopy was more sensitive than standard microscopy and culture and allowed a same day detection, but not a species identification. Positive rates were comparable for 18 S, Mucorales-PCRs, and microscopy, but the advantage of the Mucorales-PCR was a rapid detection, including species identification (within 24 h). This is informative regarding optimal antifungal therapy due to the differential intrinsic susceptibility pattern across the Mucorales species [18].

The fact that the treatment for the underlying haematological disease was suspended in around half of the patients underlines the importance of early diagnosis and management of mucormycosis to avoid both fungal infection related mortality and increased mortality due to delayed therapy of the underlying disease.

Our study has limitations. A relatively low number of patients were included from an eight year period during which new treatment modalities for the underlying diseases as well as for fungal infections (introduction of isavuconazole) have occurred. Mucormycosis is, however, a rare infection and thus, it is challenging to obtain larger case series with patients with similar underlying conditions and with a uniform approach to diagnosis and management. Other prospective studies, not restricted to haematological patients, have included 18 to 32 proven cases [21,33,34] and retrospective haematological studies of 20 to 74 proven cases during up to 20 year study periods [8,9,35,36]. We only included patients with proven mucormycosis, such as the study by Kara et al. [35]. This is both a strength (certainty of diagnosis) and a limitation. The latter because excluding cases without a proven infection may confer a risk of selection bias towards patients eligible for (invasive) sampling and subsequent therapy and thus, with a better a priori chance for survival. This concern is supported by two observations. First, a low median age among our patients was found compared to several other studies (36 years versus 40–54 years) [2,5,7,11,13,21,33,34,36]. However, patients in the only other study limited to proven cases also had a low median age of 25 years, yet reported a higher one month overall mortality rate of 55% (11/20), of which 20% (4/20) died from mucormycosis [35]. Second, a minority of our patients and those in the prior study limited to proven cases [35] had pulmonary mucormycosis, which has been associated with a higher mortality compared to sinus mucormycosis [5,7,13]. Pulmonary mucormycosis has been reported as the most common Mucorales manifestation in haematological patients [7,8,9,10,11]. However, Mucorales are ubiquitous and may be found in airway samples as contaminants, leading to an overestimation of the number of pulmonary infections when non-proven cases are included. On the other hand, the challenges associated with invasive thoracic procedures in haematological patients may lead to an underestimation of the true number of proven cases. Finally, the reported mortality is affected by the fact that all cases with mucormycosis involving CNS occurred before 2016, which is associated with a poorer outcome. However, the absence of CNS cases may also be a result of better and earlier disease control.

In conclusion, our data suggest improved treatment outcomes for mucormycosis using the following approach: An initial management including (1) early combination therapy with systemic liposomal amphotericin B 5 mg/kg and isavuconazole (with or without terbinafine), (2) imaging supported evaluation of extension and early diagnostic biopsies or immediate resection, most favourable from a sterile site with consideration of risk of surgical complications, (3) real time Blankophor microscopy of removed tissue and biopsies from resection margins (guiding subsequent surgical revisions), and (4) topical amphotericin B treatment when possible in connection with diagnostic resection, (5) dose adjustment of the antifungal treatment according to species, susceptibility pattern, and anatomic localisation (local disease vs. disseminated and CNS involvement) and TDM result, and (6) repeated surgical resections performed until radical resection is achieved. This requires a multidisciplinary approach and close collaboration between the treating physician, the surgeon, and the microbiologist providing real-time Blankophor microscopy and Mucorales-PCR guidance (within 24 h). We suggest that the optimised management principle involving early diagnosis with species identification undoubtably had a clinical impact. However, further studies of the management of this devastating infection are warranted.

## Figures and Tables

**Table 1:** Baseline clinical characteristics of the 13 patients at the time of diagnosis of the Mucorales infection.

Case #	Year	Age (Years)	Disease ^a^	Immunosuppressive Regimen ^b^	Prior Antifungal Prophylaxis (Duration Indicated for Mould Active Agents)	Regions Involved	Species
# 1	2012	26	Aplastic anaemia	Not received	None	Cerebrum	*Rhizomucor pusillus*
# 2*	2013	3	Pre-B-ALL	NOPHO ALL-protocol	FLC	Nose, sinus, orbita, and cerebrum	*Lichtheimia corymbifera*
# 3	2015	42	AML	AML-17 protocol, sorafenib followed by allogeneic HSCT. Sorafenib was initiated during fungal infection due to increasing MRD.	FLC	Lung	*Rhizopus microsporus*
# 4	2015	67	Pre-B-ALL	MD Anderson regime	PSC for three days	Sinus and orbita	*Rhizopus microsporus*
# 5	2015	28	Aplastic anaemia	ATG, ciclosporine, prednisone, and tacrolimus	PSC for approximately four months	Sinus, orbita, and cerebrum	*Rhizopus microsporus*
# 6	2016	36	Aplastic anaemia	ATG, cyclosporine, prednisone, and mycophenolate followed by two allogeneic HSCT	FLC	Sinus and near orbita	*Circinella muscae*
# 7	2016	57	AML	FLAG-Ida	PSC for three months	Lung	*Rhizopus microsporus*
# 8	2016	26	Burkitt lymphoma	R-CODOX-M	FLC	Liver	*Lichtheimia ramosa*
# 9	2017	48	Aplastic anaemia	ATG, ciclosporine, and eltrombopag	PSC for one month	Sinus and orbital wall	*Rhizopus microsporus*
# 10	2018	19	Acute T-cell lymphoblastic lymphoma	NOPHO ALL-protocol and chemotherapy intrathecally	None	The hard palate, sinus, and surrounding tissues	*Rhizopus arrhizus*
# 11	2018	60 (Lost to follow-up)	AML	FLAG-Ida	PSC for approximately 1–2 weeks	Sinus	*Rhizopus arrhizus*
# 12	2018	70	MDS RAEB-2	Azacytidine	FLC and then PSC ** for three months	Forehead	*Rhizopus microsporus*
# 13	2018	21	Diffuse large B-cell lymphoma with CNS involvement	CHOP × 3, R-CHOEP × 3, and high-dose MTX and intrathecally chemotherapy	Not prescribed	Vertebrae, lung abscess, and a subdiaphragmatic abscess	*Rhizomucor pusillus*

^a^ ALL: Acute lymphoblastic leukemia, AML: Acute myeloid leukemia, MDS: Myelodysplastic syndrome, RAEB-2: Refractory anemia with excessive blasts-2. ^b^ NOPHO: Nordic Society for Paediatric Haematology and Oncology, HSCT: Hematopoietic stem cell transplantation, MRD: Minimal residual disease, R-CODOX-M: rituximab, cycloposphamide, vincristine, doxorubicin, vincristine and methotrexate, FLAG-Ida: Fludarabine, cytarabine and granulocyte colony-stimulating factor (G-CSF) and idarubicin, R-CHOEP: Rituximab, cyclophosphamide, adriamycin, vincristine, etoposide, prednisone, MTX: Methotrexate, ATG: Antithymocyte globulin FLC: Fluconazole, PSC: Posaconazole * This case has previously been published in J Pediatr Hematol Oncol 2017; 39: e211-15, ** The therapeutic drug monitoring levels were within the recommended ranges.

**Table 2:** Overview of microbiological characteristics and diagnostic tests including all samples from each patient received at the SSI.

Case # (Year)	Species	Species Identification (Final Confirmation)	Diagnostic Tests					MIC (mg/L)			
			Blankophor Microscopy (No. Total/No. Positives)	Molecular Diagnostics (No. Total/No. Positives)			Culture No. Total/Isolates (*n* with MICs *)	AMB	PSC	ISA	TRB
				ITS	18 S	MUC-PCR					
# 1 (2012)	*Rhizomucor pusillus*	ITS isolate for species ID	2/2	Not performed			3/3 (1)	0.75	0.125	Not done	0.5
# 2 (2013) **	*Lichtheimia corymbifera*	ITS primary material	8/7	3/3			9/2 (2)	0.5–0.75	0.25	2	0.25
# 3 (2015)	*Rhizopus microsporus*	ITS and 18 S primary material	3/3	3/3	3/3		3/0				
# 4 (2015)	*Rhizopus microsporus*	ITS isolate for species ID	15/2	12/0	12/0		18/4 (4)	0.125–0.25	0.125–1	1–4	0.125–0.5
# 5 (2015)	*Rhizopus microsporus*	ITS isolate for species ID	Not performed	Not performed			1/1 (1)	0.5	0.25	2	0.5
# 6 (2016)	*Circinella* *muscae*	ITS and 18 S isolate for species ID	5/2	5/0	5/0		5/1 (1)	0.06	≤0.03	0.25	0.125
# 7 (2016)	*Rhizopus microsporus* ***	ITS primary material	3/3	4/3	3/3		4/0				
# 8 (2016)	*Lichtheimia ramosa*	18 S primary material	2/2	2/0	2/2		1/0				
# 9 (2017)	*Rhizopus microsporus*	Mucorales-PCR, ITS, and 18 S primary material	18/9	12/1	19/4	21/2	19/4 (2)	0.5–1	0.125–0.125	0.5–1	0.25–0.5
# 10 (2018)	*Rhizopus arrhizus*	Mucorales-PCR and ITS primary material	60/16	5/4	4/1	58/17	55/2 (2)	0.125	0.25	0.5–1	>4
# 11 (2018)	*Rhizopus arrhizus*	Mucorales-PCR and ITS primary material	10/9	3/2		10/10	10/8 (6)	0.25	0.25–0.5	1–2	>4
# 12 (2018) ^a^	*Rhizopus microsporus*	ITS isolate for species ID	12/2	Not performed			50/3 (1)	0.25	0.5	2	Not done
# 13 (2018)	*Rhizomucor pusillus*	Mucorales-PCR and 18 S primary material	11/5	12/4	6/3	9/5	11/0				
Cases combined for the early and late time period											
# 1–5 (2012–2015)			Median 3 (range 1–18)	Median 6 (range 0–24)			Median 3 (range 1–18)				
# 6–13 (2016–2019)			Median 10.5 (range 2–60)	Median 10 (range 0–67)			Median 10.5 (range 1–55)				

The included samples and isolates are the ones received at SSI for mycologic diagnostics either directly from the clinical department or referred from the clinical microbiological department. The MIC values are shown with ranges if more than one isolate. ITS: Internal transcribed spacer, MUC-PCR: Mucorales-PCR, AMB: Amphotericin B, ISA: Isavuconazole, PSC: Posaconazole, TRB: Terbinafine, * Cases with positive cultures only. ** This case has previously been published in J Pediatr Hematol Oncol 2017; 39: e211–15. *** The first diagnostic ITS was performed on pleurafluid at the local clinical microbiological department. ^a^ Patient diagnosed and managed in Sweden according to advice from the SSI.

**Table 3:** Diagnostic tests performed within the first 7 days from proven diagnosis.

Case # (Year)	Species	Diagnostic Tests				
		Blankophor Microscopy (No. Total/No. Positives)	Molecular Diagnostics (No. Total/No. Positives)			Culture * No. Total/Isolates
			ITS	18 S	MUC-PCR	
# 1 (2012)	*Rhizomucor pusillus*	2/2	Not performed			3/3
# 2 (2013) **	*Lichtheimia corymbifera*	Not performed	Not performed			1/1
# 3 (2015)	*Rhizopus microsporus*	3/3	3/3	3/3		3/0
# 4 (2015)	*Rhizopus microsporus*	Not performed	Not performed			1/1
# 5 (2015)	*Rhizopus microsporus*	Not performed	Not performed			1/1
# 6 (2016)	*Circinella muscae*	1/1	Nor performed			1/1
# 7 (2016)	*Rhizopus microsporus* ***	Not performed	1/1			1/0
# 8 (2016)	*Lichtheimia ramosa*	1/1		1/1		Not performed
# 9 (2017)	*Rhizopus microsporus*	7/6	5/1	6/4	7/1	7/3
# 10 (2018)	*Rhizopus arrhizus*	4/3	4/3	1/1	1/1	4/2
# 11 (2018)	*Rhizopus arrhizus*	10/9	3/2		10/10	10/8
# 12 (2018)	*Rhizopus microsporus*	8/2	Not performed			9/3
# 13 (2018)	*Rhizomucor pusillus*	2/2	3/0	2/1	2/2	2/0
In total, positive rates indicated in parenthesis		38/29 (76%) ^a^	19/10 (53%)	13/10 (77%)	20/14 (70%)	43/23 (53%) ^a^

* Culture includes isolates received from the local department for clinical microbiology. ** This case has previously been published in J Pediatr Hematol Oncol 2017; 39: e211-15. *** The first diagnostic ITS was performed directly on pleurafluid at the local clinical microbiological department. The first culture is pleura fluid. ^a^ *p* = 0.0389 comparing microscopy and culture.

**Table 4:** Systemic, topical, surgical, and supportive therapy and mucormycosis outcome of 13 haematological patients.

Case #	Systemic Antifungal Treatment, Daily Dosage	Azole Serum Concentration During Therapy *** Median (Range) (*n* Measurements)	Topical Antifungal Treatment	Surgery	Supportive Treatment	Outcome of Mucormycosis at the End of Follow Up
# 1 2012	Combination L-AMB 300 mg * × 1 and PSC 300 mg × 3 until death for approximately 2–3 weeks	s-posaconazole 1.05 (1)	Not applied	One drainage and two craniotomias	Not received	Died within one month
# 2 ** 2013	Combination L-AMB 5–9 mg/kg, PSC 37.5–75 mg/kg, and TRB 4 mg/kg for 5, 33, and 2 months, rp.	s-posaconazole 1.55 (0.42–4.73) (110)	AMB deoxycholate in the sinuses and intrathecally through Ommaya reservoir	14 resections including sinus surgeries, removal of eye, brain tissue, and hard palate	Hyperbaric oxygen 33 sessions	**Cured**
# 3 2015	Monotherapy L-AMB (3–5 mg/kg) according to kidney function and adjusted to 3 mg/kg until resection for 10 months followed by monotherapy PSC 300 mg × 1 maintenance	s-posaconazole 0.5 (0.28–1.5) (5)	Topical AMB deoxycholate in the lung cavity	One unilateral pleurapneumectomy	G-CSF	**Cured**
# 4 2015	Combination L-AMB 5 mg/kg and PSC 300 mg until death for 4 months with a few treatment interruptions	s-posaconazole 1.5 (0.0–4.2) (24)	AMB deoxycholate in the sinuses	Five resections	Not received	Died of fungal infection after four months
# 5 2015	Combination L-AMB 3.5 mg/kg for 1 month and PSC 300 mg for 2 weeks until death	Not done	Not applied	One surgical biopsy/uncinectomy and two follow-up debridements	Iron chelation in relation to iron overload	Died of fungal infection after one month
# 6 2016	Combination L-AMB 5 mg/kg daily or 10 mg/kg three times a week for 10 months. PSC 300–450 mg until death after 15 months	s-posaconazole 0.75 (0–2.4) (28)	AMB deoxycholate in the sinuses as well as inhalation	Four excisions/resections	Not received	Presumably **cured**
# 7 2016	Monotherapy L-AMB 5 mg/kg for 3 weeks and then combination of ISA 200 mg and TRB 250 mg for 4 months	Not done	Not applied	One lobectomy	G-CSF	Presumably **cured**
# 8 2016	Monotherapy PSC 300 mg for 1 month and hereafter, monotherapy ISA 200–300 mg according to TDM for 8 months	s-posaconazole 1.2 (1.1–1.6) (7) s-isavuconazole 4.8 (2.4–8.4) (22)	Not applied	Liver drainage and one resection with no hyphae	G-CSF	**Cured**
# 9 2017	L-AMB 2–5 mg/kg for four months and ISA 200 mg for 16 months. Combination was given for 3 months	s-isavuconazole 3.8 (1.4–5.7) (6)	L-AMB installations in the sinuses and intraorbitally. AMB deoxycholate inhalation. Switched to amphotericin B deoxycholate intranasally	15 resections Excluding two biopsies prior to the resections, which were inconclusive	G-CSF Granulocyte transfusions and hyperbaric oxygen approximately 11 sessions	**Cured**
# 10 2018	Combination L-AMB and ISA. AMB dosage 5–8 mg/kg initially and then reduced when fungal infection was controlled. ISA 200–600 mg for 10 months adjusted after TDM until death. Combination was given in 5 months.	s-isavuconazole 6.2 (1.2–15.4) (66)	AMB deoxycholate in the sinuses	47 interventions extending from inspection and sampling to approximately 18 surgical revisions including four larger resections	Hyperbaric oxygen 30 sessions and G-CSF	**Cured**
# 11 2018	Monotherapy L-AMB 5 mg/kg for 3 weeks and monotherapy ISA 200 mg. Duration of ISA is unknown.	Not done	AMB deoxycholate in the sinuses	Two resections	G-CSF	Presumably **Cured** (no longterm follow-up)
# 12 2018	Combination L-AMB 5 mg/kg for 3 weeks and PSC 300 mg for 3 months	s-posaconazole 1.3 (0.85–2.1) (14)	L-AMB in the wound	One resection and one skin graft	Iron chelation in relation to iron overload	**Cured**
# 13 2018	Combination L-AMB and ISA for 7 months. L-AMB dosage initially 7–10 mg/kg and then 3 mg/kg. ISA 300 mg and TRB 1–2 g/day are still ongoing.	s-isavuconazole range 3.3 (1.5–7.3) obtained August-2019 (26)	L-AMB intrathecally initially. Then changed to AMB deoxycholate	Two vertebrae resections and one lobectomy	Not received	**Cured** mucormycosis treatment is still ongoing

s: serum concentration measured in mg/L. L-AMB: Liposomal amphotericin B, AMB: Amphotericin B, TRB: Terbinafine, G-CSF: Granulocyte colony-stimulating factor. * Information regarding weight was not accessible. ** This case has previously been published in J Pediatr Hematol Oncol 2017; 39: e211–15. *** Therapeutic drug monitoring. The reported azole concentrations shown are the ones measured at Statens Serum Institute.

**Table 5:** Haematological status and survival.

Case #	Haematological Disease	Status of Haematological Disease at Fungal Infection Diagnosis and Treatment	Chemotherapy/Immunosuppression Suspended	Consequences for Haematological Course	Survival 3 Months	Survival 6 Months	Survival 12 Months
# 1 2012	Aplastic anaemia	NA	No	Yes. Unable to plan allogeneic HSCT	No	No	No
# 2 * 2013	ALL	Remission after induction	Yes	No	Yes	Yes	Yes
# 3 2015	AML	Increasing MRD	Yes	Yes. Allogeneic HSCT postponed	Yes	Yes	Yes
# 4 2015	ALL	Remission after first hyper-CVAD with rituximab and intrathecal MTX and cytarabine	Yes	Yes. Unable to proceed with chemotherapy	Yes	No	No
# 5 2015	Aplastic anaemia	Fungal infection developed during second try with ATG	Yes	Yes. Unable to proceed with immunosuppressive agents	No	No	No
# 6 2016	Aplastic anaemia	Fungal infection developed during second try with ATG	Yes	No	Yes	Yes	Yes
# 7 2016	AML	Complete remission after FLAG-ida	Yes	Yes. Prohibitive for allogeneic HSCT resulting in relapse	Yes	No	No
# 8 2016	Burkitt lymphoma	Neutropenic during the last course of chemotherapy	No	No	Yes	Yes	Yes
# 9 2017	Aplastic anaemia	Fungal infection developed after initiation of ATG	No	No	Yes	Yes	Yes
# 10 2018	Acute T-cell lymphoblastic lymphoma	Fungal infection developed during induction and intrathecal MTX and cytarabine	Yes	Yes. Later on relapse	Yes	Yes	No
# 11 2018	AML	Complete remission after FLAG-Ida	No	No	Unknown	Unknown	Unknown
# 12 2018	MDS	MDS RAEB-2 on azacythidine	No	No	Yes	Yes	Yes
# 13 2018	Diffuse large B-cell lymhoma	Remission and finalized R-CHOEP	No	No	Yes	Yes	Yes

* This case has previously been published in J Pediatr Hematol Oncol 2017; 39: e211–15. ALL: Acute lymphoblastic leukemia, AML: Acute myeloid leukemia, MDS: Myelodysplastic syndrome, NA: Not available, MRD: Minimal residual disease, CVAD: cyclophosphamide, vincristine, doxorubicine, dexamethasone, MTX: Methotrexate, ATG: Antithymocyte globulin, FLAG-Ida: Fludarabine, cytarabine and granulocyte colony-stimulating factor (G-CSF) and idarubicin, R-CHOEP: Rituximab, cyclophosphamide, Adriamycin, vincristine, etoposide and prednisone, RAEB-2: Refractory anemia with excessive blasts-2. HSCT: Haematopoetic stem cell transplantation. NA: Not available.

## Supplementary Materials

The following are available online at https://www.mdpi.com/2309-608X/6/4/268/s1, Case Presentations.
