# Supplementary material for: Introduction of a Comprehensive Diagnostic and Interdisciplinary Management Approach in Haematological Patients with Mucormycosis: A Pre and Post-Intervention Analysis
Source: J Fungi (Basel). 2020 Nov 8;6(4):268. doi: 10.3390/jof6040268 (PMC7712937; doi:10.3390/jof6040268)
Supplement: Supplementary file 1 [file jof-06-00268-s001.pdf]

Supplementary data

Case presentations

Case 1 (2012)

A 26-year old female with aplastic anaemia, but without severe neutropenia (<0.5 x 10^9/L) developed seizures and hemiparesis. CT demonstrated a large localized parietal process close to the meninges, which was partially resected by surgery. Histology of the process demonstrated non-septate hyphae, and *R. pusillus* was cultured. She was treated with liposomal amphotericin B (Liposomal-AMB) and posaconazole. Further surgical resection of the brain mucormycosis was attempted twice, but the patient died from progressive fungal infection a few weeks after.

Case 2 (2013)

A three year old girl with acute lymphoblastic leukemia (ALL) developed preseptal and infraorbital cellulitis in relation to a neutropenic phase. After a CT scan nine biopsies were sampled, of which seven were positive at microscopy, two were culture positive, and three were PCR positive (ITS PCR and sequencing) and showed *L. corymbifera*. Despite targeted posaconazole oral solution aiming a target of TDM >2 mg/L (MIC: 0.25 mg/L), the infection progressed to nasopharynx, orbit and brain shown at an MR scan. Extensive surgery was performed involving removal of part of the brain tissue, orbita, hard palate and a hemimaxillectomy. The patient recovered after surgery and triple-compound (liposomal-AMB, posaconazole and terbinafine) systemic antifungal treatment, amphotericin B deoxycholate intranasally (50 mg/L of sterile water solution) during surgeries and intrathecally (5 mg/mL sterile water solution) repeatedly. This case has been published previously (22).

Case 3 (2015)

A 42-year old male carpenter treated for acute myeloid leukaemia (AML) developed severe neutropenia and pneumonia. A CT scan raised suspicion of invasive pulmonary aspergillosis in the right lung. However, microscopy, culture, *Aspergillus* galactomannan antigen and ITS PCR and sequencing of bronchopulmonary lavage (BAL) was unable to confirm fungal infection. He was empirically treated with liposomal-AMB for seven weeks before an extensive pleurapneumonectomy with local amphotericin B deoxycholate (100 mg/L) installation was done. Pauciseptate hyphae was found by microscopy, and *R. microsporus* was confirmed by ITS PCR and sequencing. Liposomal-AMB treatment systemically continued at a dosage of 3-5 mg/kg (adjusted during treatment according to GFR) during and after subsequent allogeneic haematopoietic stem cell transplantation (allo-HSCT). Liposomal-AMB was terminated seven months after

allo-HSCT. The patient fully recovered and continued on posaconazole up until today, 5 years after allo-HSCT, as he still receives immunosuppressive agents.

Case 4 (2015)

A 67-year old female was diagnosed with Philadelphia negative acute lymphoblastic leukaemia (ALL) and treated with hyper fractionated cyclophosphamide, vincristine, adriamycin, dexamethasone (hyper-CVAD) and rituximab together with intrathecal methotrexate (MTX) and cytarabine. After nine days of severe neutropenia, she developed signs of severe rhino-orbital invasive fungal infection shown at a CT scan of the facial skeletal. A total of five extensive surgical resections of fungal infected tissues was performed to radically remove affected areas in the sinus, conchae, septum and periorbital surrounding together with a hemi-maxillectomy. Resections were recurrently culture positive for *R. microsporus* with ITS PCR and sequencing confirming the species identification. At the last biopsies (day 113) microscopy was positive, but negative by means of ITS and 18S PCR and culture. None of the performed diagnostic ITS and 18S PCRs directly on the biopsies were positive. The patient was treated with liposomal-AMB 5 mg/kg and posaconazole. Daily local amphotericin B deoxycholate installations at a dosage of 50 mg in 100 mL sterile water for 2.5 months were also administered. Her ALL was in remission three months after debut, but her performance did not allow further cytotoxic chemotherapy. The patient died from fungal infection two days after the last surgery.

Case 5 (2015)

28-year old male with prolonged hospitalisation for aplastic anaemia, treated with multiple blood cell transfusions and iron chelation. Three months after initiation of immunosuppressive therapy and long-term neutropenia the patient developed pain near sinus and the eye. A CT scan showed blurred sinus ethmoidalis, sinus maxillaris, periorbital inflammation and exophthalmus. An ethmoidectomy was performed with opening to sinus maxillaris and orbital decompression. Two days later the patient developed generalized seizures and was admitted to the intensive care unit. Therefore a CT and an MR scan of the brain were performed, which showed a 2 x 2 x 4 cm hypodense process basofrontally. *R. microsporus* was later grown from the maxillary sinus. The patient was treated with liposomal-AMB 3.5 mg/kg and posaconazole tablets 300 mg daily. The patient was ineligible for further surgery and died five weeks after the surgical procedure.

Case 6 (2016)

A 36-year old male with aplastic anaemia developed sinusitis during severe long-term neutropenia. A Positron Emissions Tomography (PET) CT scan showed uptake involving the maxillary sinuses. A functional

endoscopic sinus surgery (FESS) was performed with opening of sinus maxillares. Histopathologic examination of biopsy confirmed the presence of hyaline pauci-septate hyphae and associated tissue damage, and culture was positive. Species was molecularly identified as *C. muscae*. He was treated with liposomal-AMB systemically (5 mg/kg/day), amphotericin B locally 250-300 mg/L with 10 mL applied in the nose daily and with posaconazole 300 mg intravenously daily. The patient underwent allo-HSCT, but rejected the graft and underwent a second allo-HSCT. CT scan showed continuously sinus blur with discrete involvement of the orbita wall. Further surgical debridements were performed with local amphotericin B installations, but exenteration was not performed. *Acremonium pinkertoniae* and *Saccharomyces cerevisiae* were detected after surgical debridement, but *C. muscae* was not retrieved at the three following excisions/resections, and he was presumably cured for mucormycosis. The prolonged high dose amphotericin B (ten months) resulted in kidney failure and dialysis. The patient died from multiple complications in relation to his long hospital stay 15 months after the diagnosis of *Mucorales* infection.

Case 7 (2016)

A 57-year old male with AML and refractory disease developed pulmonary infiltration during neutropenia after chemotherapy. The patient received empirical antifungal treatment initially with caspofungin and then voriconazole. *R. microsporus* was identified by means of ITS PCR and sequencing of pleura fluid five to six weeks after the pleuradrainage. The patient started liposomal-AMB 5 mg/kg/day, but did not tolerate it and had increasing pulmonary infitrations. Afterwards he started isavuconazole as the first patient in Denmark and terbinafine. After considering the surgical risks the patient underwent a lobectomy with abscess drainage three months after the pleuradrainage. The surgical resect also confirmed the fungal diagnosis by means of microscopy and ITS PCR and sequencing. Due to the fungal infection the patient's AML progressed since he could not receive cytotoxic chemotherapy. Ruxolitinib was initiated experimentally, but did not have an effect on his AML. The patient died from AML 2.5 months after the surgical procedure.

Case 8 (2016)

26-year old female with Burkitt lymphoma developed neutropenic fever after the final rituximab, cycloposphamide, vincristine, doxorubicin, vincristine and methotrexate (R-CODOX-M). A CT scan showed a 3.5 cm process in the right liver lobe. Due to suspected aspergillosis, empirical caspofungin treatment was changed to voriconazole. A liver biopsy demonstrated hyphae at microscopy at the department of pathology. Molecular analysis confirmed *L. ramosa* by 18S DNA analysis. The therapy was switched to first posaconazole orally and then isavuconazole for eight months. Surgical liver drainage was performed, with confirmation of *L. ramosa* by 18S DNA and microscopy, but culture was negative. At follow-up an

ultrasound showed a process of 3.5 cm in the liver. A resection was executed, but tissue examination did not show any hyphae. The patient recovered.

Case 9 (2017)

A 48-year old male with severe aplastic anaemia and persistent severe neutropenia complained of pain from the jaw. A CT scan showed blurred sinuses, but no cerebral involvement. Prophylactic posaconazole was changed to liposomal-AMB 3 mg/kg. Surgical tissue sampling was performed involving an ethmoidectomy and drainage from sinus maxillaris. Species identification was conclusive on the third surgical resection. *R. microsporus* was identified by means of microscopy, culture, Mucorales-PCR, ITS and 18S PCR and sequencing. After species identification liposomal-AMB was increased to 5 mg/day. A total of 17 ear-nose-throat surgical interventions were done to ensure radical debridement, which included resection of the orbital wall. There was extensive invasive tissue involvement of the maxillary and ethmoid sinus and the surroundings of the right eye. Further, *Aspergillus fumigatus* and *Aspergillus flavus* were also recurrently identified from the affected tissue. The last two surgical interventions were without resections since no necrosis was detected. The patient was treated with systemic liposomal-AMB, isavuconazole and local liposomal-AMB installations in sinus and 1 mL liposomal-AMB intraorbitally and in the fatty tissue sorrounding the eye (3.5 mg/mL) in connection to surgery together with amphotericin B deoxycholate inhalation 25 mg twice a day. The patient switched to amphotericin B deoxycholate locally in the sinuses. Adjunctive granulocyte transfusions and hyperbaric oxygen were also given. The patient fully recovered.

Case 10 (2018)

19-year old male with lymphoblast T-cell lymphoma (LBL) was treated after the Nordic Society of Pediatric Haematology and Oncology (NOPHO) regime including chemotherapy intrathecally. Three weeks after the initiation of cytotoxic chemotherapy prior to the first neutropenic phase the patient developed dark discolouration with progressive necrosis in the hard palate and was started on liposomal-AMB 5 mg/kg/day and i.v. isavuconazole. A biopsy showed *R. arrhizus*. Two out of 55 initial and control cultures were positive, and 16 out of 60 microscopies were with pauci-septate hyphae. The patient underwent 47 ear-nose-throat interventions. The surgical interventions extended from inspection and biopsy to excessive surgery involving the hard palate and a unilateral hemi-maxillectomy followed by frequent supplementary debridements, which were guided by microscopy and Mucorales-PCR of the resection margins. Sampling in relation to eye surroundings was also performed, but no fungal involvement was detected. Approximately 18 surgical resections were performed with local installations with amphotericin B deoxycholate. Amphotericin B deoxycholate (250 mg/L) 5 mL was also supplemented three times a day locally until fungal control and then dose adjusted to 5 mg/L to avoid tissue reaction applying a volume of 100-200 mL in the

nose three times a day. The exposed bone was covered during palatal reconstruction with a free flap after several mycosis negative debridements. Amphotericin B continued for five months overall. Isavuconazole dosing were adjusted according to TDM up to a dosage of 600 mg daily. Initially a serum concentration of isavuconazole (s-isavuconazole) >2 mg/L was preferred, then >8 mg/L until negative diagnostics followed by a maintenance concentration of >2 mg/L. The patient recovered from his fungal infection, but died from his underlying disease 10 months after the diagnosis of his fungal infection.

Case 11 (2018)

A 60-years old female (from foreign country) was treated for relapse AML and went into complete remission. During her last neutropenic episode she developed sinus soreness, and liposomal-AMB was initiated. Excisions were performed twice from the involved necrotic tissue from concha inferior and media and the anterior ethmoid, and the patient received local installations with amphotericin B deoxycholate (10 mg/L) three times a day. *R. arrhizus* was detected by means of microscopy, culture, Mucorales-PCR and ITS PCR and sequencing. Surgical excisions were performed along with neutrophilic regeneration. The infection resolved and no signs of fungal infection were present objectively, but not confirmed by microscopy or molecularly. The patient received three weeks of liposomal-MB overall and started isavuconazole as she departed one week after her mucormycosis diagnosis. Long-term follow-up data are not accessible.

Case 12 (2018)

A 70-year old male with myelodysplastic syndrome (MDS) and persistent neutropenia developed a necrotic wound on his forehead in a hematoma after falling. The wound progressed from 4 to 35 mm in diameter in two days. Culture yielded growth of *R. microsporus*, which was molecularly confirmed with ITS PCR and sequencing. Broad pauci septate fungal hyphae were abundant in scraped wound material by Blankophor direct microscopy, confirming cutaneous mycormycosis. A CT scan of the skull did not reveal any signs of bone involvement. Systemic liposomal-AMB 5 mg/kg was initiated, and his posaconazole prophylaxis was changed to therapeutic drug monitored treatment. Two days after diagnosis the mucormycosis was surgically removed down to the periost in one piece and with one cm lateral margin. One biopsy close to the lesion (2 mm) was positive in culture and microscopy. Fungal culture and Blankophor staining of biopsies around and under the removed mucormycosis were negative for growth or fungal structures. Histopathology with periodic acid–Schiff stain and Grocott methenamine silver stain confirmed that the mucormycosis had been radically removed with a 2 mm margin in depth and a wide lateral margin. Daily fungal wound cultures and Blankophor direct microscopy were performed the first week and were all negative. A total of 50 samples were collected, of which two out of twelve were positive at microscopy and three out of 50 cultures were positive. The post-operative wound was topically treated with L-AMB (200

mg/L) daily flushes and soaked compresses. Split skin grafting was applied three weeks after surgery and topical L-AMB continued for another week. Although the patient was persistently neutropenic, the wound healed completely without relapse.

Case 13 (2018)

21-year old male with Stage IV diffuse large B-cell lymphoma with pulmonary involvement. After 4 months of treatment with R-CHOEP with intra-thecal MTX he achieved complete remission. One month after the last treatment, he developed severe debilitating lumbar back pain. PET-CT and MRI of the spine demonstrated severe L3-L5 spondylodiscitis with arachnoiditis and spinal stenosis together with a subphrenic process and a lung process CT guided vertebral biopsy was microscopy, Mucorales-PCR and 18S positive. *R. pusillus* was determined by Mucorales-PCR and 18S analysis and was also detected in biopsy from the subphrenic liver abscess through Mucorales-PCR, ITS PCR and sequencing. The patient was treated with liposomal-AMB at a dosage of 7 to 10 mg/kg initially with kidney function monitoring, isavuconazole 300 mg (increased dosage) and terbinafine 500 mg twice a day. Spinal fluid was microscopy, culture and PCR negative for *Mucorales.* Surgical removal of vertebrae L4 *in toto* and debridement of the extensive adjacent necrotic fungal destructed tissues was stabilized with spondylodesis and insertion of cage. The vertebrae showed pauci-septate hyphae at microscopy, and PCR was positive. During surgery amphotericin B was installed locally at a dosage of 50 mg, and a catheter was installed for subsequent amphotericin B installations intrathecally. Liposomal-AMB dosage was reduced to 3 mg/kg due to decreased renal clearance and isavuconazole was adjusted to target a s-isavuconazole >2 mg/L. Hospitalisation was complicated by hospital/surgically related bacterial infections, partial lower limb paresis and severe nerve pain secondary to extensive arachnoiditis. Response evaluation was done by close PET-CT, which demonstrated satisfactory regression of the lumbar changes, but relatively unchanged size and PET-activity of the lung and liver abscesses. Six months after diagnosis a lobectomy was performed and *R. pusillus* was identified from the inside of the resect by means of Mucorales-PCR, but not retrieved by culture or microscopy. Surgical removal of the subphrenic liver abscess had too high risk. After 7 months of liposomal-AMB treatment in combination with isavuconazole and terbinafine PET-CT demonstrated further regression of the subphrenic process and regression of activity at the lumbar spine. Currently, one year after hospital discharge, he remains in remission from lymphoma and mucormycosis, during continued well-tolerated isavuconazole and terbinafine treatment. There is now only a small liver PET-positive liver lesion.
